# Supplementary material for: Ontogenetic Variation in Macrocyclic and Hemicyclic Poplar Rust Fungi
Source: Int J Mol Sci. 2022 Oct 27;23(21):13062. doi: 10.3390/ijms232113062 (PMC9653582; doi:10.3390/ijms232113062)

Supplementary

Table S1. Open reading frame of *STE3.3* and *STE3.4* genes in *Melampsora* spp.

Name	ORF length	Amino acid number	GenBank ID
MlpSTE3.3-URE	1158	385	MW363516
MlpSTE3.3-TEL	1158	385	MW927156
MmedSTE3.3-URE	1158	385	MW927144
MmedSTE3.3-TEL	1161	386	MW927159
MpruSTE3.3-URE	1152	383	MW927145
MpruSTE3.3-TEL	1152	383	MW927157
MlpSTE3.4-URE	1245	414	MW363515
MlpSTE3.4-TEL	1245	414	MW927160
MmedSTE3.4-URE	1245	414	MW927147
MmedSTE3.4-TEL	1245	414	MW927163
MpruSTE3.4-URE	1212	403	MW927149
MpruSTE3.4-TEL	1212	403	MW927161
MmagSTE3.4-URE	1212	403	MW927148
MmagSTE3.4-TEL	1212	403	MW927162

Table S2. Predicted of physical and chemical properties of STE3.3 and STE3.4 protein in *Melampsora* spp.

Name	Molecular weight	Theoretical pI	Formula	Instability index (II)	Average Hydropathy
MlpSTE3.3-URE	43128.73	8.97	$C_{1981}H_{3090}N_{516}O_{520}S_{21}$	36.83	0.392
MlpSTE3.3-TEL	43046.67	8.97	$C_{1974}H_{3092}N_{516}O_{520}S_{21}$	37.09	0.399
MmedSTE3.3-URE	43054.61	8.97	$C_{1974}H_{3084}N_{516}O_{521}S_{21}$	36.05	0.384
MmedSTE3.3-TEL	43185.78	8.97	$C_{1983}H_{3093}N_{517}O_{521}S_{21}$	36.76	0.397
MpruSTE3.3-URE	43135.58	8.78	$C_{1974}H_{3067}N_{515}O_{524}S_{23}$	35.33	0.363
MpruSTE3.3-TEL	43135.58	8.78	$C_{1974}H_{3067}N_{515}O_{524}S_{23}$	35.33	0.363
MlpSTE3.4-URE	45766.19	8.86	$C_{2075}H_{3223}N_{549}O_{571}S_{24}$	30.60	0.282
MlpSTE3.4-TEL	45766.19	8.86	$C_{2075}H_{3223}N_{549}O_{571}S_{24}$	30.60	0.282
MmedSTE3.4-URE	45766.19	8.86	$C_{2075}H_{3223}N_{549}O_{571}S_{24}$	30.60	0.282
MmedSTE3.4-TEL	45814.28	8.93	$C_{2080}H_{3227}N_{549}O_{570}S_{24}$	31.17	0.288
MpruSTE3.4-URE	44985.61	8.96	$C_{2063}H_{3199}N_{535}O_{551}S_{21}$	37.62	0.325
MpruSTE3.4-TEL	44989.60	8.96	$C_{2062}H_{3199}N_{535}O_{552}S_{21}$	36.39	0.326
MmagSTE3.4-URE	45085.68	8.96	$C_{2070}H_{3199}N_{535}O_{552}S_{21}$	35.18	0.325
MmagSTE3.4-TEL	45051.71	9.03	$C_{2068}H_{3205}N_{535}O_{551}S_{21}$	35.70	0.335

Table S3. Hydrophilicity, hydrophobicity and transmembrane structure of STE3.3 and STE3.4 proteins in *Melampsora* spp.

Sequences	Hydrophobicity peak	Hydrophilicity peak	Transmembrane helix	Number of helical amino acids in membrane	Probability of N-terminal on one side of plasma membrane
MlpSTE3.3-URE	Position:130 Score:2.800(max)	Position:252 Score:-2.400(min)	7	153.85225	9.31%
MlpSTE3.3-TEL			7	153.85564	9.31%
MmedSTE3.3-URE			7	153.85235	9.31%
MmedSTE3.3-TEL	Position:131 Score:2.800(max)	Position:253 Score:-2.400(min)	7	154.47249	7.65%
MpruSTE3.3-URE	Position:130 Score:2.800(max)	Position:252 Score:-2.400(min)	7	153.82699	11.18%
MpruSTE3.3-TEL			7	153.82699	11.18%
MlpSTE3.4-URE	Position:281 Score:2.989(max)	Position:386 Score:-2.300(min)	6	135.50797	84.80%
MlpSTE3.4-TEL			6	135.50797	84.80%
MmedSTE3.4-URE			6	135.50797	84.80%
MmedSTE3.4-TEL			6	134.85116	87.83%
MpruSTE3.4-URE	Position:281 Score:3.333(max)	Position:108 Score:-2.544(min)	6	140.20469	70.39%
MpruSTE3.4-TEL			6	141.6942	62.59%
MmagSTE3.4-URE			6	141.71101	62.59%
MmagSTE3.4-TEL			6	141.68069	62.59%

Table S4. Subcellular localization analysis of STE3.3 and STE3.4 protein in *Melampsora* spp.

Sequences	Endoplasmic reticulum	Mitochondria	acuole	Cytoplasm	Nucleus	Vesicles	Golgi apparatus
MlpSTE3.3-URE	66.7%	33.3%	/	/	/	/	/
MlpSTE3.3-TEL	66.7%	33.3%	/	/	/	/	/
MmedSTE3.3-URE	66.7%	33.3%	/	/	/	/	/
MmedSTE3.3-TEL	66.7%	33.3%	/	/	/	/	/
MpruSTE3.3-URE	66.7%	33.3%	/	/	/	/	/
MpruSTE3.3-TEL	66.7%	33.3%	/	/	/	/	/
MlpSTE3.4-URE	33.3%	11.1%	11.1%	11.1%	11.1%	11.1%	11.1%
MlpSTE3.4-TEL	33.3%	11.1%	11.1%	11.1%	11.1%	11.1%	11.1%
MmedSTE3.4-URE	33.3%	11.1%	11.1%	11.1%	11.1%	11.1%	11.1%
MmedSTE3.4-TEL	33.3%	11.1%	11.1%	11.1%	11.1%	11.1%	11.1%
MpruSTE3.4-URE	77.8%	22.2%	/	/	/	/	/
MpruSTE3.4-TEL	66.7%	22.2%	/	/	11.1%	/	/
MmagSTE3.4-URE	66.7%	22.2%	/	/	11.1%	/	/
MmagSTE3.4-TEL	66.7%	22.2%	/	/	11.1%	/	/

Note: “/” indicates that it does not exist.

Table S5. Signal peptide of STE3.3 and STE3.4 protein in *Melampsora* spp.

Sequences	Division value D	Signal peptide sequence
MlpSTE3.3-URE	0.578	1-26
MlpSTE3.3-TEL	0.578	1-26
MmedSTE3.3-URE	0.578	1-26
MmedSTE3.3-TEL	0.578	1-26
MpruSTE3.3-URE	0.584	1-18
MpruSTE3.3-TEL	0.584	1-18
MlpSTE3.4-URE	0.509	1-17
MlpSTE3.4-TEL	0.509	1-17
MmedSTE3.4-URE	0.509	1-17
MmedSTE3.4-TEL	0.509	1-17
MpruSTE3.4-URE	0.516	1-17
MpruSTE3.4-TEL	0.516	1-17
MmagSTE3.4-URE	0.516	1-17
MmagSTE3.4-TEL	0.516	1-17

Supplemental Figures

Figure S1. The tertiary structure of STE3.3 and STE3.4 protein based on Mlp

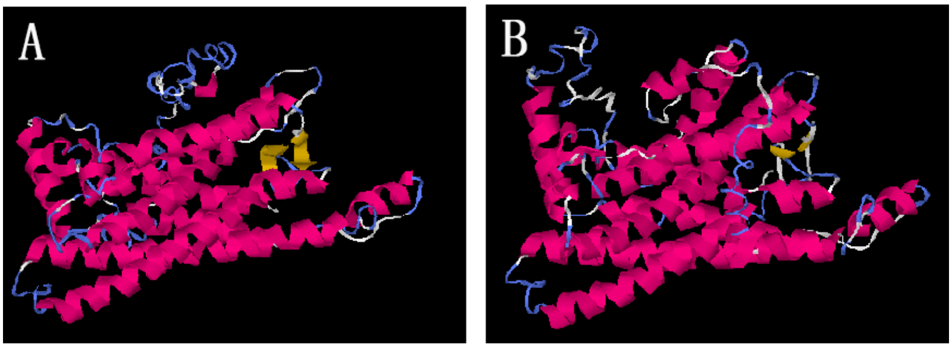

Figure S2. Nuclei of basidiospore under fluorescence microcopy

Note: A-C, Mlp; D-F, Mmed; A&D, Germinated teliospore; B&F, Probasidium; C&F, Basdiospora

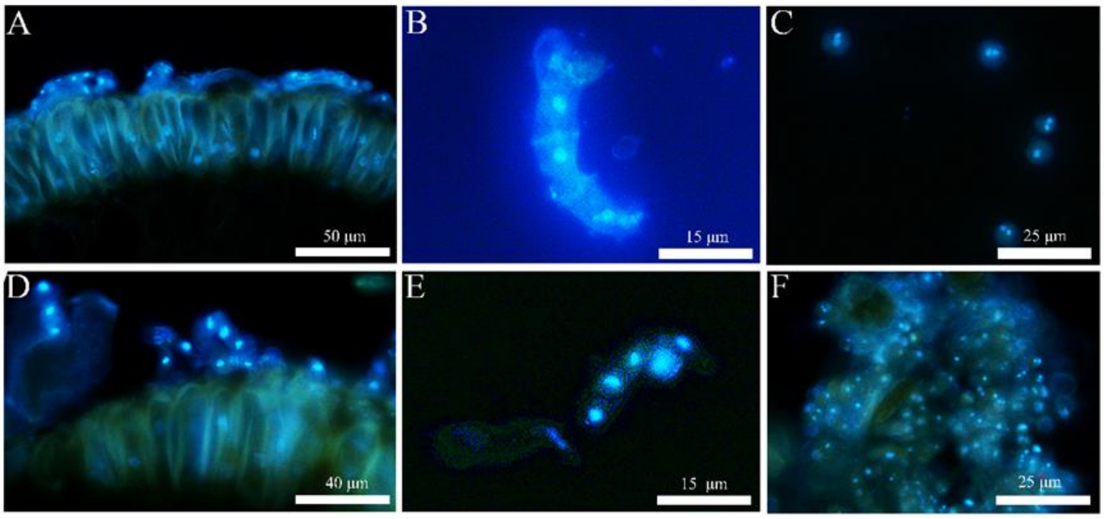

Figure S3. Morphological characteristics of pycnia and aecia observed by transmission electron microscope (TEM)

Note: A, Ontogeny of aecia, the below under the line is monokaryotic, but the above is dikaryotic; B, Dicaryotization by cell membrane fusion

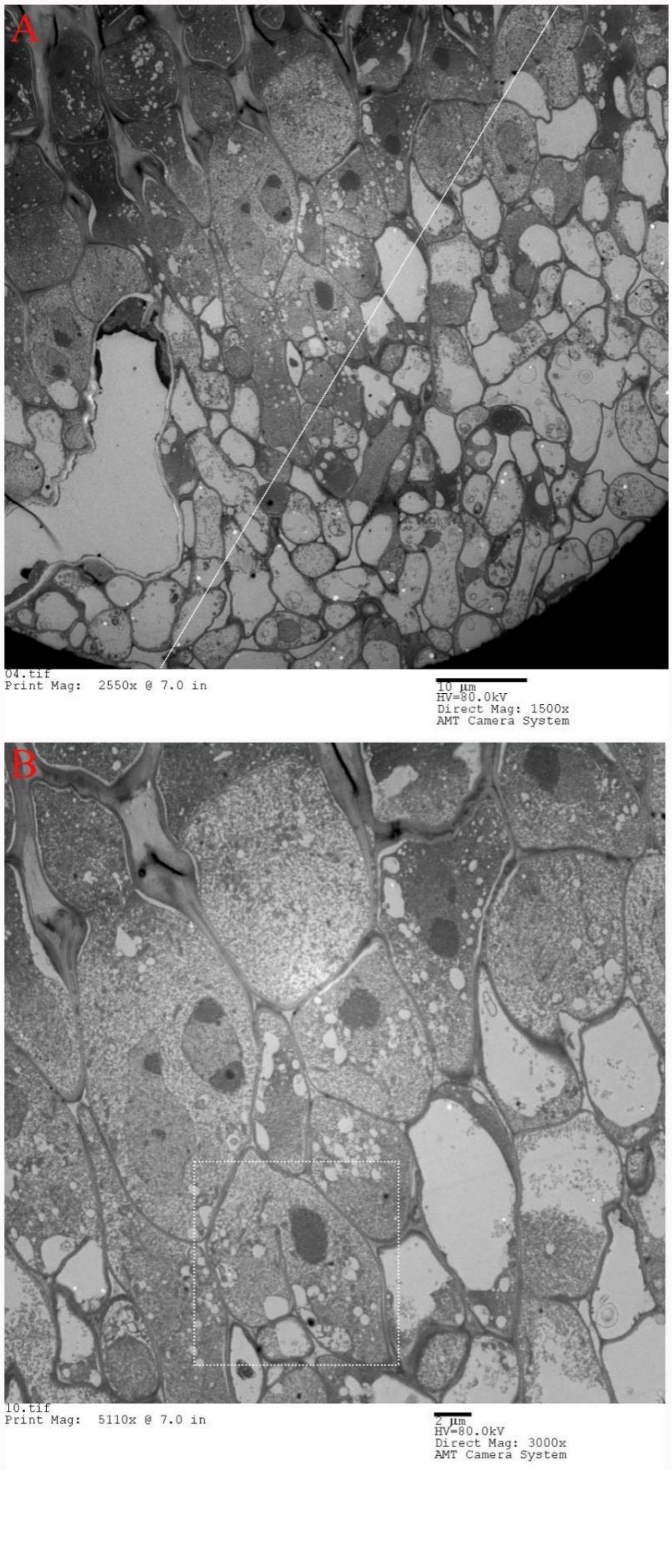

Figure S4. Buds of poplar collected in this study

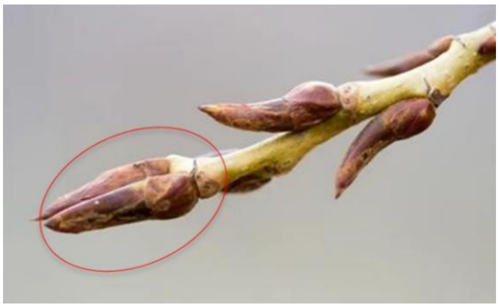

Supplement: Supplementary file 1 [file ijms-23-13062-s001.zip › ijms-1962661-supplementary.pdf]
